# Digital inequalities in the sharing economy: A qualitative text analysis from focus groups

**DOI:** 10.1371/journal.pone.0357784

**Published:** 2026-09-11

**Authors:** David Coy-Mesa, Yilsy Núñez Guerrero, Gustavo Morales-Alonso

**Affiliations:** Department of Organization Engineering, Business Administration, and Statistics, Universidad Politécnica de Madrid, Madrid, Spain; IULM: Libera Universita di Lingue e Comunicazione, ITALY

## Abstract

The sharing economy has transformed access to services through digital platforms and the way people consume them. However, participation remains uneven owing to persistent digital inequalities. This study examines how digital skills and access conditions shape individuals’ participation, with particular emphasis on how users perceive and experience these inequalities. A qualitative approach was adopted, based on two focus groups involving 14 participants aged 25–64, conducted in Madrid, Spain, in July 2024. The analysis followed the Gioia methodology and was complemented by exploratory Natural Language Processing techniques to identify recurring terms and named entities in the transcripts. The findings reveal five interrelated dimensions shaping user engagement with platforms such as Uber, Airbnb, and Booking: usability, technology, skills, trust, and other usage limitations. These dimensions show how limited digital skills, usability challenges, access conditions, security concerns, and additional socioeconomic constraints can restrict participation, particularly among users with fewer digital resources or lower levels of digital competence. The study contributes an empirically grounded, user-centered framework for understanding how multiple digital barriers accumulate and shape unequal participation in the sharing economy. It also connects digital inequality research with platform participation by showing that exclusion results not only from limited Internet access or skills, but also from platform design, trust, accessibility, and payment conditions. These findings provide actionable insights for researchers, platform designers, policymakers, and educators seeking to promote more inclusive sharing economy platforms.

## 1. Introduction

Technological evolution has been a major factor in the diversification of collaborative economy platforms in recent years, which has had a significant global impact [1]. The sharing economy (SE) is characterized by the exchange of goods between individuals, better use of underutilized resources, temporary access to services and, in particular, the coordination of supply and demand through digital platforms [2]. Airbnb and Uber were the pioneering platforms that have revolutionized the accommodation and transportation sectors by changing consumption patterns. Today, this platform model extends to many other sectors such as fashion, banking and logistics [3].

Despite this growth, participation remains uneven. A report by the European Commission data [4] showed that the use of content-sharing platforms in Europe rose from 17% to 23% between 2016 and 2018, although adoption is concentrated among urban, younger and more highly educated populations. The digital divide reflects broader social inequalities and will persist as long as these structural differences remain [5,6].

Citizens with stronger digital skills and greater familiarity with digital technologies tend to view digitalization more positively and express greater confidence in new technologies, whereas limited skills can hinder full participation [7]. In this context, digital literacy is essential not only for navigating digital environments but also for understanding and effectively using digital information. These capabilities are necessary for meaningful participation in contemporary digital society [8].

The literature indicates that digital skills and the frequency of internet searches influence participation in the sharing economy [9]. Other factors, such as educational attainment and social capital, may also shape the intensity of platform use [10]. Although previous studies have identified sociodemographic patterns associated with participation in digital environments, less is known about how individuals experience these barriers in their everyday use of digital platforms, particularly how they perceive the challenges they face.

This study addresses this gap by examining how digital skills and access conditions shape participation in the sharing economy, with particular attention to how users perceive and experience digital inequalities when using digital platforms. Accordingly, the study addresses the following research question: How do individuals perceive and experience digital inequalities in their participation in the sharing economy, particularly those related to digital skills and access barriers?

This study contributes to the literature by providing an empirically grounded, user-centered account of how digital inequalities shape participation in the sharing economy. Rather than examining digital access, skills, usability, trust, and socioeconomic constraints in isolation, it shows how these barriers interact and accumulate across users’ experiences with digital platforms. By combining the Gioia methodology with complementary exploratory text analysis, the study also provides a transparent qualitative analysis of the barriers that may limit individuals’ ability to access and effectively use sharing economy services. The resulting five-dimensional framework advances current discussions of digital inequality by linking individual digital resources to platform design and the broader conditions that shape participation.

The remainder of the paper is structured as follows. Section 2 reviews the literature on the sharing economy, digital inequalities, digital skills, and barriers to platform participation. Section 3 describes the research design, participant recruitment, data collection, Gioia analysis, and complementary NLP procedures. Section 4 presents the results of the qualitative coding and the exploratory text analysis. Section 5 discusses the findings in relation to the existing literature. Finally, Section 6 presents the conclusions, practical implications, limitations, and directions for future research.

## 2. Literature review

### 2.1. Sharing economy

Sharing economy (SE) platforms have diversified considerably in recent years, attracting growing attention from academics, policymakers, and practitioners [11]. The concept has been described using various terms, including the platform economy, the sharing economy, on-demand platforms, the access economy, and the peer-to-peer economy [12], reflecting the expansion and complexity of the phenomenon [13]. The SE can be defined as the coordination of exchanges among individuals to acquire and distribute resources in return for compensation, which may be financial or nonfinancial [14]. This business model generally operates through online platforms that allow individuals to bypass traditional commercial channels and share underutilized resources, thereby reducing transaction costs [15].

The literature generally recognizes that the sharing economy generates economic, social, and environmental impacts that can contribute to sustainable development [16].

In terms of economic impacts, the SE fosters entrepreneurship, job creation and growth, with profit being the main motivation for participation [17,18]. Furthermore, cost reduction plays a key role in participation [19].

From a social perspective, the sharing economy can foster relationships within communities [20]. It may also facilitate access to services and opportunities for exchange while creating social bonds among participants [21]. The rapid development of the internet, mobile devices, and artificial intelligence has played an important role in the growth of sharing economy platforms [22].

However, digitalization has not led to equal participation across population groups. Access to and effective use of these platforms depend not only on technological infrastructure but also on users’ digital skills and resources. This disparity reflects the broader issue of digital inequality, which is discussed in the following section.

### 2.2. Digital divide and skills

The digital divide refers to disparities in the ability of individuals and groups to access and effectively use digital technologies [23]. These disparities can reinforce socioeconomic inequalities and limit employment opportunities worldwide. Their effects may be particularly significant for young people, who may face exclusion from the labor market and limited access to the educational opportunities needed to participate in an increasingly digitalized economy [24].

Van Dijk [25] distinguishes three cumulative levels of the digital divide. The first-level digital divide (1995–2003) concerns physical access to hardware and internet connectivity. The second-level digital divide (2004–present) focuses on differences in digital skills and patterns of use. This distinction, introduced by Hargittai [26], moves beyond binary inequalities in access to consider differences in what people do online. The third-level digital divide, identified from 2012 onward, concerns differences in the extent to which internet use translates into positive offline outcomes [27]. In technologically advanced countries, fewer than 10% of the population lack basic internet access [25].

In this context, digital competencies are considered a key component of the second-level digital divide and can be defined as the skills required to use digital technologies effectively in everyday life [28]. Related terms, such as digital skills and digital literacy, are often used interchangeably to describe similar capabilities [29].

The European Commission defines digital competence as the safe, critical, and responsible use of and engagement with digital technologies for learning, work, and participation in society. It encompasses information and data literacy, communication and collaboration, media literacy, digital content creation, safety—including digital well-being and cybersecurity—an understanding of intellectual property, problem-solving, and critical thinking [30].

As digital technologies become increasingly central to economic and social participation, differences in digital skills can shape people’s ability to benefit from the opportunities they offer. These differences may reinforce existing inequalities, particularly in the context of SE platforms, where participation depends not only on access but also on the skills needed to use these platforms effectively.

### 2.3. Digital divide and skills in the sharing economy

Despite the growing body of research on digital inequalities and the sharing economy, the intersection between these two fields remains relatively underexplored. Existing studies have focused primarily on transportation and accommodation platforms, using methods such as interviews, case studies, index-based analyses, and national surveys. Although a limited number of studies have examined digital skills, most of the literature focuses on the effects of digital literacy on workers and companies that provide these services.

Early evidence showed how basic access barriers can limit participation in the sharing economy. Dillahunt et al. [31] documented cases in which individuals using basic mobile phones without internet access were unable to use Uber, illustrating how the digital divide can translate into exclusion from sharing economy services. Quirós et al. [32] found that advanced digital skills and regular e-commerce use were associated with a greater likelihood of using Uber, while Erazo et al. [33] found that gender and income were associated with different patterns of platform use in the accommodation sector.

These findings suggest that digital access, skills, and socioeconomic conditions are associated with how individuals participate in specific sharing economy platforms. However, a fuller understanding of these inequalities also requires consideration of the broader operational and service-related conditions that shape shared mobility systems, particularly car-sharing services.

Turoń [34] identifies 151 quantitative and qualitative criteria affecting car-sharing services during their implementation and operation and classifies them into six groups: economic and technical, transportation, social, environmental, organizational, and other factors. These criteria cover organizational conditions, the characteristics of current and potential users, service costs, transportation infrastructure, and the demographic and social structure of cities [34]. Although this framework provides a comprehensive overview of the conditions affecting car-sharing systems, it focuses mainly on their implementation and operation. Digital skills are not explicitly considered as a separate factor or category. The criterion most closely related to digital inequality is the “accessibility of the IT system for the user,” which is classified as an organizational factor. This limitation highlights the need to complement system-level analyses with a user-level perspective that considers digital skills and inequalities in platform access and use.

At the individual level, other studies have examined the factors associated with the adoption of shared mobility services. In the context of on-demand ride services, Alemi et al. [35] conducted an empirical study using the California Millennials Dataset, collected through an online survey of California residents in fall 2015. The sample included millennials aged 18–34 and members of Generation X aged 35–50 from six regions of the state, including urban, suburban, and rural areas. The findings showed that services such as Uber and Lyft were more likely to be adopted by older millennials—that is, individuals aged 25–34—and by those with at least a bachelor’s degree. Adoption was also associated with previous experience using taxi and car-sharing services, frequent smartphone use for transportation-related decisions, and more positive attitudes toward technology. These findings suggest that greater familiarity with digital technologies in everyday life was associated with a higher likelihood of adopting these services [35].

More recent research has shown that barriers to shared mobility are perceived differently across population groups, including both users and nonusers. Chahine et al. [36] conducted an online survey of adult residents of Indianapolis to examine perceptions of ride-hailing, bike-sharing, and shared e-scooter services. Their analysis considered two perceived benefits—improved connectivity with public transit and reduced parking demand—and two perceived barriers: service cost and technology-related difficulties. The largest groups reported predominantly neutral or indifferent views, suggesting that a substantial share of the population had not fully embraced shared mobility services. In contrast, a smaller “barrier-conscious” group indicated that lower costs and fewer technology-related obstacles could encourage greater use. Across most of the identified groups, safety and reliability were also considered important factors when choosing a mode of transportation [36]. These differences in the perceived benefits and barriers of shared mobility are consistent with broader patterns of participation in the sharing economy.

At a broader level, Lutz et al. [9] found that demographic factors, including age, gender, education, income, and household size, as well as psychological factors such as trust, digital skills, and the frequency of online searches, are important predictors of participation in SE platforms. Previous research has also identified significant differences across population groups in the types and range of activities performed online [37]. In particular, socioeconomically advantaged individuals tend to use the internet more frequently for information-intensive and capital-enhancing activities, whereas less advantaged users are more likely to use it for recreational purposes, such as entertainment [38].

These patterns are also reflected in participation in the sharing economy. Because such participation is mediated by digital technologies and often requires users to search for and evaluate information, it is positively associated with online search skills and behaviors [9]. More recent research by Lutz et al. [39], also identified differences by age and place of residence, with younger urban residents perceiving short-term rental platforms as more beneficial than older and rural residents.

Eichhorn et al. [10] examine digital inequality in a commercial setting, focusing on participation in SE platforms. They analyze how structural social conditions shape different stages of platform access and use. Their findings indicate that van Dijk’s resource-based model of internet use [40] can be applied to explain online participation in the sharing economy.

### 2.4. Research gap

Although empirical research has established a link between digital inequalities and platform participation, important gaps remain. Much of the existing literature has relied primarily on quantitative analyses, providing limited insight into users’ subjective experiences and perceptions. As a result, the roles of digital skills, internet access, and platform usability have not been examined in sufficient depth from a user-centered perspective.

This study addresses these gaps by exploring how digital skills and access conditions shape individual participation in the sharing economy and how individuals perceive and experience inequalities in platform-based environments. To guide the empirical analysis, the study draws on a set of research propositions derived from the literature reviewed:

- First, limited digital skills and restricted internet access are perceived as significant barriers to effective participation in sharing economy platforms.
- Second, economically disadvantaged individuals face greater barriers to accessing and benefiting from sharing economy services because of limited digital resources.
- Third, digital skills, internet use, and the frequency of online searches are considered important conditions for effective participation in the sharing economy.
- Finally, users identify specific usability and accessibility challenges in existing sharing economy platforms that may hinder broader and more inclusive participation.

## 3. Materials and methods

### 3.1. Data collection

Focus groups were conducted to capture a range of perspectives on the research topic and to understand the issues from the participants’ perspectives. The group setting facilitates the collection of diverse views within a single session [41]. Fern [42] found that a single focus group can identify approximately 70% of the themes that could be obtained from an equivalent number of in-depth interviews, highlighting the efficiency of this method for initial conceptual exploration. The study did not seek statistical representativeness; rather, it aimed to achieve theoretical richness and analytical sufficiency.

Given the exploratory nature of the study, we adopted a qualitative approach to gain an in-depth understanding of individuals’ perceptions of participation in sharing economy platforms. Focus group discussions were conducted using a semi-structured guide organized around four themes: the role of digital skills; access to and barriers within the sharing economy; the social and economic implications of digital barriers; and proposals for improving platform accessibility and inclusivity. The study was guided by an interpretivist perspective, focusing on participants’ subjective experiences and the meanings they attributed to their interactions with sharing economy platforms.

Consequently, two focus groups were conducted, each with seven participants. The same semi-structured discussion guide, consisting of open-ended questions, was used in both sessions (see S1 File) to encourage participants to express their views. The corresponding author reviewed and coded the two transcripts sequentially using the Gioia methodology, beginning with FG1 and then proceeding to FG2. The coding process was documented in an Excel matrix that linked each excerpt to its focus group, participant, first-order concept, second-order theme, and aggregate dimension. The other two co-authors then reviewed the coding. Before combining the data for reporting, the three authors compared the coding structures derived from the two groups. The analysis of FG2 yielded the same five aggregate dimensions identified in FG1 and did not produce any additional higher-order dimensions. Although FG2 provided further examples, nuances, and second-order themes, these were incorporated into the existing dimensional structure. Drawing on Hennink et al.’s [43] distinction between code saturation and meaning saturation, and considering the stability of the five aggregate dimensions across both groups, the three authors jointly concluded that saturation had been reached at the level of the aggregate dimensional structure. After reviewing FG2, they therefore decided that a third focus group was not necessary. The data were considered sufficient to address the exploratory research question at this analytical level, without claiming that meaning saturation had been achieved.

The focus groups were led by a facilitator who guided the discussions, while another researcher served as an observer, monitoring group dynamics and taking notes. All participants provided written informed consent before the sessions began. With appropriate measures in place to protect confidentiality, both sessions were audio-recorded, yielding a total of 45 pages of transcribed material. The Ethics Committee for R&D + I Activities at the Universidad Politécnica de Madrid determined that the study did not require Institutional Review Board review or approval because no personal data were used in the research.

### 3.2. Selection of participants

Fourteen adults participated in two focus groups conducted in July 2024. Participants were recruited between June 3 and July 14, 2024, using a combination of purposive and convenience sampling. The corresponding author contacted potential participants by email and WhatsApp through professional and institutional networks. Before providing consent, invitees received general information about the study topic and purpose, the voluntary nature of their involvement, and the recording of the sessions. The information provided was intentionally broad to avoid influencing their responses during the focus groups.

Eligible participants were between 25 and 64 years of age and resided in Madrid, Spain, at the time of data collection. The lower age limit was established because the study focused on adults who were currently or potentially active in economic and social contexts and who had varying levels of exposure to sharing economy platforms. The upper age limit reflected the initial scope of the recruitment strategy rather than an assumption that adults over 64 were unaffected by digital barriers. Participants had either current or previous experience using at least one sharing economy platform or had personally encountered barriers that prevented them from using such platforms.

Recruitment sought variation in levels of digital proficiency, ranging from novice to advanced users, as well as diversity in participants’ occupational and educational backgrounds; participants included individuals with experience in business administration, technology education, digital skills training, and academia. Despite this strategy, the final group included a comparatively high proportion of participants with higher education and professional experience. The findings should therefore not be interpreted as representative of platform users or nonusers in Madrid or Spain. Rather, the study was designed to provide exploratory qualitative insights into how usability, access to technology, digital skills, trust, and other usage limitations may shape participation in sharing economy platforms.

Participants attended one of the two sessions based on their availability on the scheduled dates. Both groups included individuals with varying levels of digital experience and diverse professional backgrounds, and the data from both sessions were analyzed jointly. Table 1 summarizes the participants’ main characteristics and the criteria supporting their inclusion in the study.

**Table 1:** Characteristics of the participants.

Focus Group	ID	Gender	Age Range	Occupation	Selection criteria
FG1	1	Female	35-44 years	e-learning	Professional digital experience
FG1	2	Other	25-34 years	PhD Student	Academic and research profile
FG1	3	Male	35-44 years	Unemployed	Economically vulnerable user
FG1	4	Female	45-54 years	Civil Servant	Institutional digital user
FG1	5	Male	35-44 years	Researcher	Digital competence experience
FG1	6	Female	25-34 years	Librarian	Information access professional
FG1	7	Male	45-54 years	Teacher	Digital skills educator
FG2	8	Female	55-64 years	Education Entrepreneur	Older user within an entrepreneurial context
FG2	9	Female	45-54 years	Senior Industrial Engineer	Technical or industrial digital user
FG2	10	Female	35-44 years	Teacher	Education sector
FG2	11	Female	35-44 years	European Projects Technician	Institutional or political digital user
FG2	12	Female	25-34 years	Student	Digital native/ younger generation
FG2	13	Female	25-34 years	Student	Digital native/ younger generation
FG2	14	Male	35-44 years	Engineer	Technical professional

### 3.3. Data analysis

The discussions were transcribed and carefully reviewed to correct transcription and spelling errors and remove filler words. Participants’ identities were anonymized by replacing their names with unique research codes in the format FGx-Pi, where x indicates the focus group number and i identifies the participant. The data were then analyzed using the Gioia methodology, complemented by natural language processing (NLP) techniques as an exploratory analytical tool. This combination supported a structured interpretation of participants’ perspectives while providing additional exploratory insights into recurring patterns in the textual data.

#### 3.3.1. Gioia Methodology.

The primary data analysis was conducted using the Gioia methodology, which provides a structured approach to inductive qualitative research [44]. This methodology involves organizing the data into first-order concepts, second-order themes, and aggregate dimensions. Coding is a central part of the analytical process and involves systematically reviewing and interpreting field notes, transcripts, or other qualitative materials while preserving the relationships among the different parts of the data [45].

Both focus group transcripts were first read in full to develop an overall understanding of the discussions. Coding was then conducted sequentially, beginning with FG1 and followed by FG2. Relevant passages were identified as units of meaning and assigned first-order labels that remained close to the participants’ own words and the meanings they conveyed, in accordance with the Gioia methodology [44]. This process yielded 330 first-order concepts. The coding was documented in an Excel matrix that recorded the focus group, participant identifier, original quotation, first-order concept, second-order theme, and aggregate dimension associated with each excerpt. This procedure maintained clear traceability between the original data and each level of the final analytical structure.

In the next phase of the analysis, the first-order concepts were compared and grouped into 64 second-order themes. This process involved examining similarities, differences, and recurring patterns across the first-order concepts to organize them into a more manageable set of categories. The emerging themes were then evaluated according to their ability to describe and explain the phenomena reflected in participants’ accounts [44]. To support this process, pivot tables were created for each emerging dimension. These tables facilitated the systematic organization and comparison of related concepts, the identification of overlaps and redundancies, and the assessment of coherence within each grouping. They were not used to automate the coding process but rather as an analytical support tool that allowed the researchers to examine conceptually similar labels together and refine the boundaries and names of the second-order themes. When necessary, overlapping labels were merged, and coded excerpts were reassigned to the theme that most accurately represented their meaning.

Finally, the second-order themes were grouped into five aggregate dimensions, providing a structured interpretation of the data. These dimensions were usability, technology, skills, trust, and other usage limitations. The resulting analytical structure was used to develop a data structure diagram that visually represents the relationships among the first-order concepts, second-order themes, and aggregate dimensions.

The initial coding was conducted by the corresponding author. The preliminary coding and emerging data structure were then reviewed by the other two co-authors. This collaborative review covered the grouping of first-order concepts, the formulation and naming of second-order themes, and the definition of the aggregate dimensions. Differences in the interpretation, naming, or classification of concepts and themes were discussed jointly and resolved by consensus. The analytical structure was refined through this iterative process until all three authors agreed that it adequately reflected the participants’ accounts and was conceptually coherent across the different levels of analysis.

The initial coding was conducted by the corresponding author. The preliminary coding and emerging data structure were then reviewed by the other two co-authors. This collaborative review examined the grouping of first-order concepts, the formulation and naming of second-order themes, and the definition of the aggregate dimensions. Differences in the interpretation, naming, or classification of concepts and themes were discussed jointly and resolved by consensus. The analytical structure was refined through this iterative process until all three authors agreed that it adequately reflected the participants’ accounts and was conceptually coherent across the different levels of analysis.

The final data structure was reviewed by tracing the analytical progression from the original participant quotations to the first-order concepts, second-order themes, and aggregate dimensions; examining the relationships among the three analytical levels; and comparing the coding structures of FG1 and FG2 before combining the data from both groups for reporting. FG2 contributed additional examples, nuances, and second-order themes, all of which were incorporated into the five-dimensional structure identified in FG1 and did not require the development of an additional aggregate dimension. Table 2 provides illustrative examples of how participant quotations informed the development of first-order concepts, second-order themes, and aggregate dimensions.

**Table 2:** Illustrative examples of the coding structure (from quotation to aggregate dimension).

Representative quotation	First-order concept	Second-order theme	Aggregate dimension
“Now there are apps that, as well as having transport services, also have a marketplace. They have financial services and so everything becomes a little more complex” (FG1, P6).	The shift toward multifunctional platforms.	Adaptability and sustainability of business models on digital platforms	Usability
“We’ve all had that experience of traveling to a remote village, to some unknown place and finding that there is no coverage; it feels as though you are lost” (FG2, P2).	Coverage issues in remote areas.	Access by region	Technology
“There is an age-related digital divide because I think young people have greater access, simply because they navigate these environments better” (FG1, P6).	Young people’s ease with technology.	Intergenerational digital literacy challenges	Skills
“I am more afraid now than before because the negative experiences of friends and family are more widely known” (FG1, P3).	Perception of risk and fear.	Privacy and security	Trust
“How can a blind person shop on Wallapop? How can I book an Uber if I’m blind or hard of hearing?” (FG2, P4).	Experiences of people with disabilities.	Accessibility	Other usage limitations

#### 3.3.2. Natural language processing (NLP).

In addition to the qualitative coding conducted using the Gioia methodology, natural language processing (NLP) techniques were applied as a complementary exploratory tool. The analysis provided a descriptive overview of the language used in the focus group discussions by identifying recurring words, frequently occurring lemmas, and named entities. The NLP analysis was not intended to replace the interpretive qualitative analysis or to statistically validate the findings.

The Spanish-language transcript corpus was processed using Python 3.12.8, the spaCy library, and the Spanish model es_core_news_md. The spaCy pipeline supports several linguistic processing tasks, including tokenization, part-of-speech tagging, syntactic parsing, lemmatization, and named entity recognition [46]. The analysis specifically used tokenization, stopword removal, lemmatization, and NER.

For the word-frequency analysis, all tokens were converted to lowercase. Spanish stopwords were removed using the default stopword classification provided by the spaCy model through the “token.is_stop” attribute. Punctuation, numeric tokens, and whitespace tokens were also excluded. The remaining tokens were counted to identify the most frequent words in the corpus.

The same general filtering criteria were applied to the lemma-frequency analysis. The lowercase lemma of each retained token was obtained using the “token.lemma” attribute, allowing inflected forms of the same word to be grouped together. No part-of-speech filtering was applied. The resulting frequencies were exported and used to generate the corresponding visualizations. Generic terms related to the organization of the sessions, such as “participant,” “topic,” “question,” and “example,” were excluded from the final visualizations when they did not contribute meaningfully to the interpretation of the discussions.

Named entity recognition was used to identify references to organizations and geographic locations. Entities were automatically extracted from the processed corpus using spaCy’s “doc.ents” attribute. The analysis focused primarily on entities classified as organizations (ORG) and locations (LOC), including sharing economy platforms, countries, cities, and other geographic references mentioned during the discussions. The extracted entities were then manually reviewed to remove false positives and correct platform names that had been omitted or assigned to an incorrect entity category.

Finally, word frequencies, lemma frequencies, and named entities were summarized using frequency plots and word clouds. For presentation in the English-language manuscript, selected Spanish words, lemmas, and geographic names were manually translated into English, while platform names were retained in their original form. The original frequency counts were preserved. The NLP results were interpreted alongside the findings from the Gioia analysis to provide an additional descriptive perspective on the language used by participants. They indicate which terms and entities occurred repeatedly in this specific corpus but were not treated as evidence of statistical significance, thematic relevance, or independent validation of the qualitative findings.

## 4. Results

### 4.1. Dimensions of user experience on sharing economy platforms

**Fig 1:**
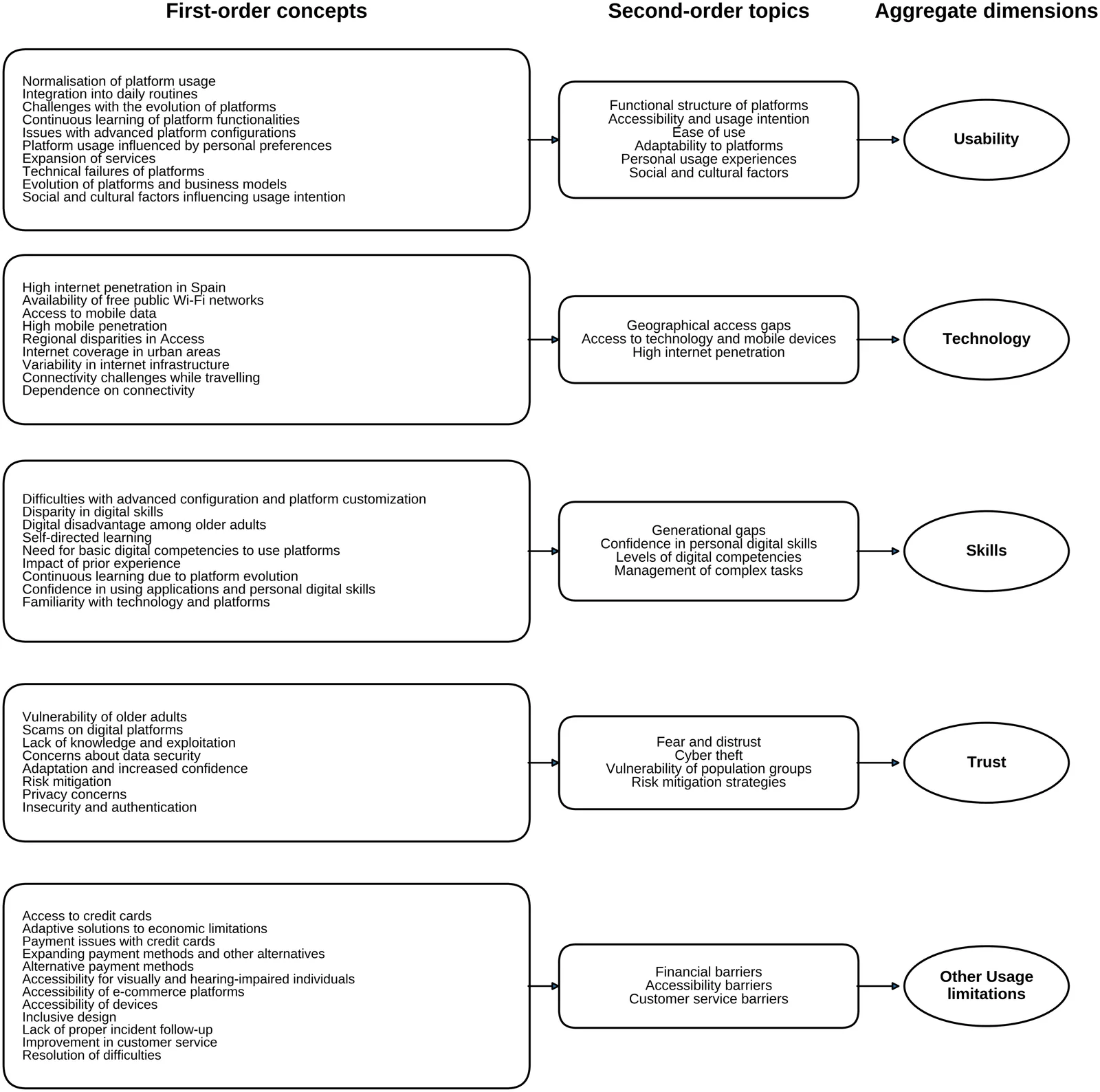
Data structure diagram using the Gioia Method.

The analysis of the focus group discussions identified five aggregate dimensions: usability, technology, skills, trust, and other usage limitations. Fig 1 presents the data structure developed following the Gioia methodology, including the first-order concepts, second-order themes, and aggregate dimensions.

#### 4.1.1. Usability.

Participants emphasized the importance of user experience, particularly the simplicity and intuitiveness of platform interfaces. For example, one participant stated that “*they are all very intuitive*” (FG1, P4), while another commented that “*they are very easy to use*” (FG2, P2). Although some participants mentioned technical obstacles, they also described becoming more familiar with the platforms after overcoming initial difficulties. However, the growing range of services offered within a single application was perceived as a potential source of complexity. One participant explained, “*Some apps now offer not only transportation services but also a marketplace and financial services, which makes everything a little more complex*” (FG1, P6).

The discussions also suggested that adapting to sharing economy platforms may require an ongoing learning process. Participants referred particularly to potential difficulties among older adults, including limited digital literacy, unfamiliarity with digital services, and the continued use of basic or outdated devices. These observations reflect participants’ perceptions, as adults aged 65 or older were not represented in the sample. For example, one participant noted, “*I think older adults are falling a little behind despite their interest*” (FG2, P6). The same participant suggested that some older adults continue to use basic or outdated phones because they do not feel a need to adopt newer devices, which may further widen the digital divide: “*Older people who feel they* *don’t need it use old and basic phones*” (FG2, P6). Advanced platform settings were also perceived as potentially more difficult for some users and were described as “*much more challenging*” (FG2, P4).

Individual preferences and social influences were also described as factors shaping the use or nonuse of sharing economy platforms. Some participants preferred other options or continued to rely on traditional services, whereas others sought information before deciding whether to adopt a platform. For example, one participant noted, “*I am interested in learning more about it before deciding whether I want to use it*” (FG2, P2). Participants also referred to the influence of opinions and experiences shared within their social circles, which could either encourage or discourage platform use. One participant explained, “*I have never used Airbnb, although I know about it from comments made by close friends*” (FG1, P7).

#### 4.1.2. Technology.

Participants identified connectivity as an important factor shaping access to and use of SE platforms. Some recalled experiencing substantial internet access difficulties in the past, although they generally perceived that these barriers had become less common in Spain because of the wider availability of mobile data plans and public Wi-Fi networks. At the same time, participants noted that connectivity inequalities may persist in rural and remote areas, where network coverage is often more limited. These observations reflect participants’ experiences and perceptions, as rural residents were not represented in the sample. For example, one participant explained, “*We have all experienced traveling to a remote village or an unfamiliar place and finding that there is no coverage; it feels as though you are lost*” (FG2, P2).

Participants also reported that connectivity problems may occur in urban settings, particularly in densely populated or crowded areas. They also perceived that the availability of some platform-based services varies across geographic markets, as providers may be less likely to operate in areas with lower demand or limited commercial viability. For example, one participant remarked, “*Some platforms may not be viable in certain locations*” (FG1, P4).

The discussions also highlighted the potential role of economic constraints in limiting access to digital devices and connectivity. Some participants referred to individuals who may be unable to afford a smartphone or computer, although those experiencing the most severe forms of material and digital exclusion were not represented in the focus groups. For example, one participant stated, “*There are also people who cannot afford to buy a smartphone*” (FG2, P6). Participants further suggested that limited financial resources may restrict access to mobile data plans and, consequently, to SE platforms, even though connectivity costs were perceived to have declined in recent years. As one participant noted, “*Financial resources also limit access to these platforms*” (FG1, P5).

Finally, participants described differences in internet infrastructure and connectivity across countries. Some referred to unstable connections and high international roaming costs when traveling outside Spain, suggesting that access to platform-based services may also depend on the technological and economic conditions of a particular location.

#### 4.1.3. Skills.

The findings highlighted varied experiences and perceptions related to digital skills and adaptation to technology. Participants frequently referred to age-related differences in familiarity with digital environments, suggesting that younger users may face fewer difficulties because they have been exposed to digital technologies from an earlier age. For example, one participant stated, “*There is an age-related digital divide because I think younger people have greater access, simply because they are better able to navigate these environments*” (FG1, P6). Participants also perceived that older adults may need more time, support, and opportunities to adapt to digital platforms. These statements reflect participants’ perceptions, as adults aged 65 or older were not included in the sample. One participant noted, “*I think developing digital literacy is more difficult at certain ages than at others*” (FG1, P1). Some participants illustrated this perception by referring to relatives who had difficulty using smartphones and preferred simpler devices: “*Some parents get confused when using smartphones and cannot manage them, so they have to continue using old button phones*” (FG2, P7).

At the same time, participants did not portray digital adaptation as fixed or determined solely by age. They described cases in which individuals gradually developed digital skills through continued exposure and practice. Several participants also expressed confidence in their own digital abilities and indicated that limited digital skills had not prevented them from using SE platforms. As one participant stated, “*Regarding digital competence, I do not consider it to have been a limitation in my case*” (FG1, P6).

The continuous evolution of digital platforms was described as requiring an ongoing learning process. Participants emphasized the need to adapt as digital tools and platform features change. As one participant explained, “*I do not think it is a matter of already having all the necessary skills, but rather of continuing to learn as the tools evolve*” (FG1, P1). This learning was described as taking place through different pathways, including self-directed experimentation and formal or workplace training. One participant stated, “*I taught myself by exploring, experimenting, making mistakes, and learning through trial and error*” (FG1, P7). Other participants referred to professional settings in which intermediate or advanced digital skills were either developed or required.

Participants generally considered a minimum level of digital competence necessary to access and use SE platforms. However, they also distinguished between basic use and more advanced features. Personalization options, account settings, and increasingly complex interfaces were perceived as additional challenges, particularly for users with less experience or confidence. The discussions therefore suggest that barriers may arise not only from limited basic skills but also from the increasing complexity of the tasks involved in using some platforms.

#### 4.1.4. Trust.

This dimension captured recurring concerns about disclosing personal information and the perceived risk of fraud or data theft when using SE platforms. These concerns were particularly associated with online payments and requests for personal data. For example, one participant stated, “*What limits me is the mistrust I feel when entering personal information*” (FG1, P6). Participants also referred to negative experiences reported by friends and relatives, which appeared to reinforce their own caution toward platform use. As one participant explained, “*I am* *more concerned now than before because I hear more about the negative experiences of friends and family”* (FG1, P3).

At the same time, some participants described trust as something that could develop gradually through repeated use and perceptions of improved platform security. However, concerns persisted when platforms requested information that participants considered excessive or particularly sensitive, such as home addresses or identification details. For example, one participant stated, “*When they start asking for too much personal information, I become reluctant to provide it*” (FG2, P2).

Participants also expressed concern about older adults’ potential vulnerability to scams and fraudulent practices. These observations were generally based on experiences involving relatives or acquaintances rather than on accounts provided directly by older adults. For example, one participant noted, “*I am thinking particularly of older people and speaking from my parents’ experience, as this has happened to them before. I think they are more vulnerable to scams when using these platforms*” (FG2, P3). These statements should therefore be interpreted as participants’ perceptions of age-related vulnerability rather than as direct evidence from older platform users.

Finally, participants described several strategies for reducing perceived risks while continuing to use sharing economy platforms. These included using credit cards with low spending limits and avoiding public Wi-Fi networks when conducting transactions. For example, one participant stated, “*I have a card with a very low credit limit that I use only for bookings because I am very concerned about the risks*” (FG1, P7). These strategies illustrate how users attempt to balance their concerns with their desire to benefit from the opportunities offered by sharing economy platforms.

#### 4.1.5. Other usage limitations.

Participants also identified other important barriers not captured by the previous dimensions, including financial constraints, accessibility issues, and limitations in customer service. Dependence on credit cards was described as an obstacle because not all individuals have access to one, and some participants noted that cards issued in other countries may not be accepted. For example, one participant stated, “*Many platforms require a credit card, which creates a financial barrier because people without access to one cannot use many of these services*” (FG1, P2). However, some participants acknowledged the availability of alternatives, such as cash payments, and suggested that expanding payment options could promote inclusion and help reduce the digital divide.

Participants also raised concerns about the accessibility of SE platforms for people with visual or hearing impairments. These observations reflected participants’ perceptions and examples rather than the direct experiences of people with disabilities, who were not specifically recruited for the study. For example, one participant asked, “*How can a blind person shop on Wallapop? How can I book an Uber if I am blind or hard of hearing?*” (FG2, P4). Although some participants perceived that certain platforms already offered accessibility features, they believed that further improvements were needed to accommodate a broader range of users.

In addition, shortcomings in customer support emerged as another important concern. Participants expressed dissatisfaction with automated support systems, such as chatbots and automated telephone services, and placed greater value on human assistance. For example, one participant stated, “*Sometimes you have a question, but it is impossible to get a useful answer from the chatbot*” (FG2, P6). Participants also described difficulties in following up on incidents and obtaining timely solutions, suggesting that inadequate customer support may negatively affect the overall platform experience.

Taken together, the five dimensions suggest that unequal participation in the sharing economy does not result from a single barrier. Instead, participants described an accumulation of difficulties related to platform usability, access to technology, digital skills, trust, and other conditions of use. These dimensions provide the main interpretive framework for the findings. The complementary NLP analysis presented in the following section offers an additional descriptive perspective on the corpus by identifying the words, lemmas, platform names, and geographic references that appeared repeatedly across the discussions.

### 4.2. Complementary text analysis using NLP

**Fig 2:**
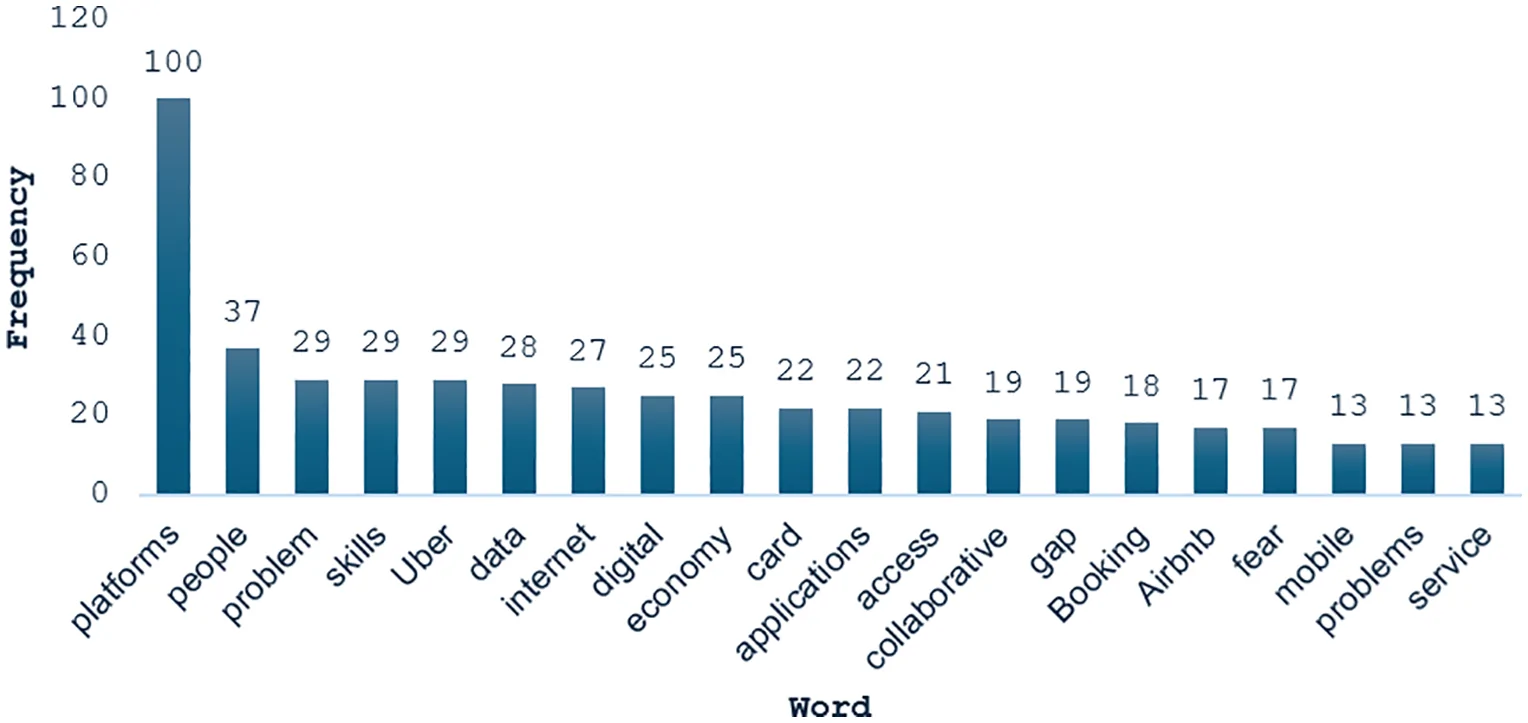
Most frequent terms in the focus group transcripts.

**Fig 3:**
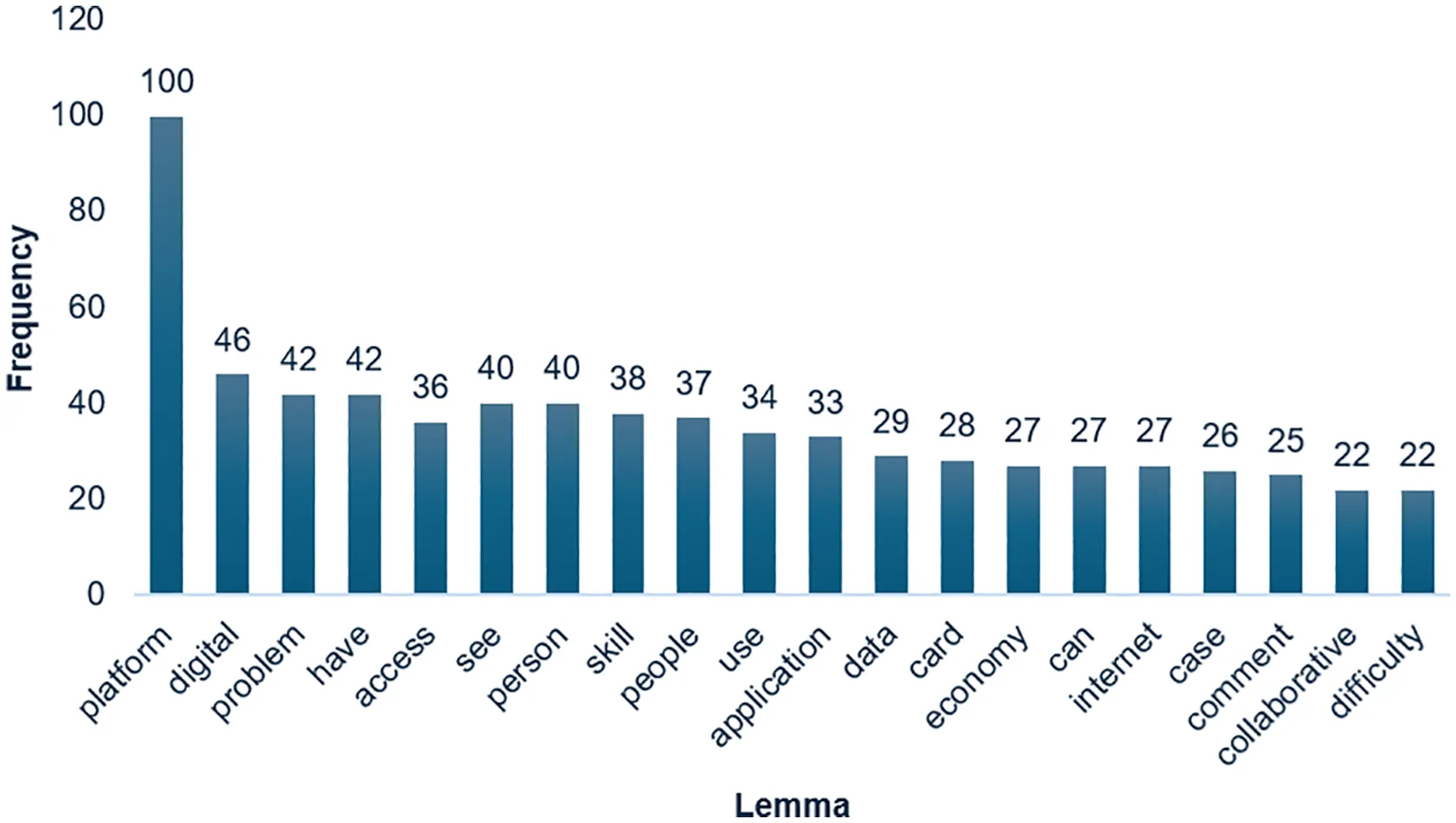
Most frequent lemmas in the focus group transcripts.

**Fig 4:**
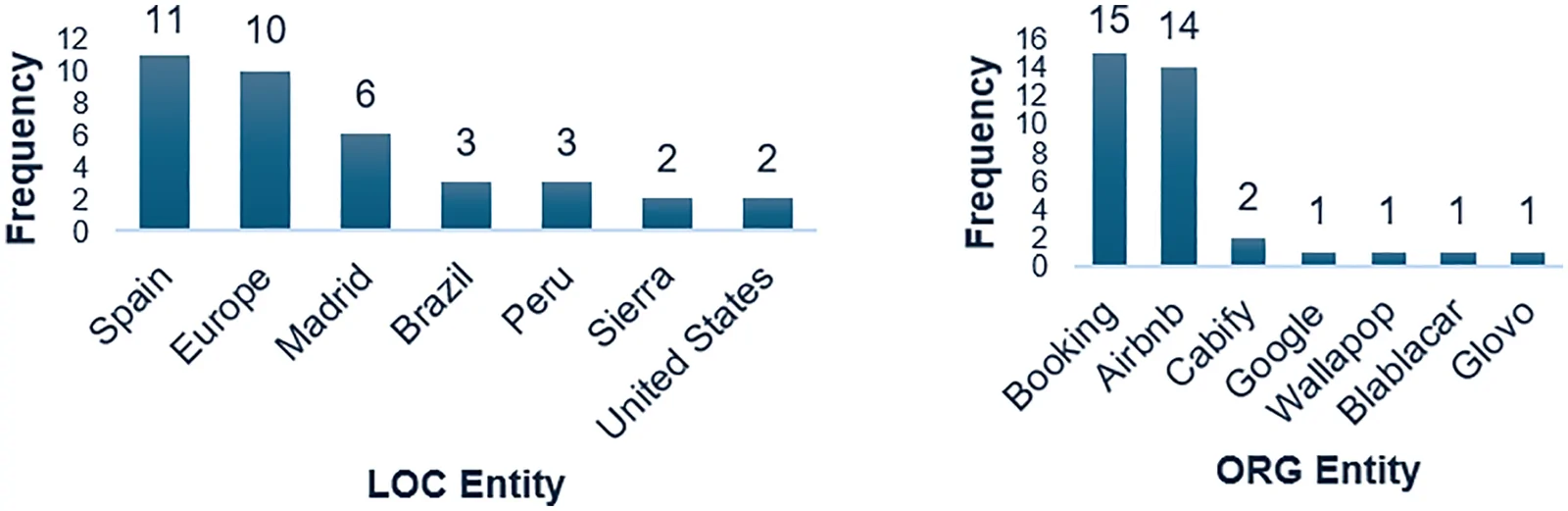
Most frequent named entities.

Building on the qualitative findings presented in the previous section, word-frequency analysis, lemma-frequency analysis, and named entity recognition (NER) were used as complementary exploratory procedures to examine recurring lexical patterns in the focus group transcripts. The five dimensions identified through the Gioia analysis provided the primary interpretive framework, while the NLP analysis offered an additional, transparent overview of the words, lemmas, platform names, and geographic references that appeared repeatedly in the discussions. These procedures were not intended to statistically validate the qualitative findings but rather to make recurring elements of the textual corpus more visible. The results are presented in bar charts showing the most frequent words in the transcripts (Fig 2), the most frequent lemmas obtained through lemmatization (Fig 3), and the most frequent named entities classified as locations (LOC) and organizations (ORG) (Fig 4).

Fig 2 shows the most frequent words in the focus group transcripts. The most frequent word was “platforms,” with 100 occurrences, followed by “people” [37], “skills” [29], “problem” [29], “Uber” [29], “data” [28], “internet” [27], “digital” [25], “economy” [25], and “card” [22]. The recurrence of these words was consistent with several issues identified in the qualitative analysis. The terms “skills,” “digital,” and “internet” appeared in participants’ accounts of digital competence and access conditions, while “data” was frequently associated with concerns about trust and the disclosure of personal information. References to “card” appeared mainly in discussions of payment requirements and financial barriers, whereas “economy” was commonly used in references to the sharing economy.

The frequent occurrence of words such as “platforms” and “problem” indicates that digital platforms and difficulties associated with their use appeared repeatedly in the discussions. Participants frequently referred to platforms such as Uber, Booking, and Airbnb when describing their experiences, often in connection with usability, access, and the digital divide. Similarly, the recurrence of “skills” was consistent with participants’ accounts, in which digital competence emerged as an important condition for accessing and using these platforms.

Fig 3 presents the most frequent lemmas identified through the NLP analysis. Lemmatization groups inflected word forms under their corresponding base forms. The most frequent lemma was “platform,” with 100 occurrences, followed by “digital” [46], “problem” [42], “has” [42], “see” [40], “person” [40], “skill” [38], “people” [37], “use” [34], and “application” [33]. The recurrence of lemmas related to platforms, individuals, skills, use, and problems indicates that these elements appeared repeatedly in participants’ discussions of platform participation. This lexical pattern was consistent with the qualitative accounts of usability and digital skills barriers. However, the frequency counts alone do not establish relationships among the terms or independently confirm the qualitative dimensions.

Fig 4 presents the most frequent named entities classified as locations (LOC) and organizations (ORG). The location entities included references to Spain, Europe, Madrid, Brazil, and Peru. These places served as contextual reference points when participants described or compared experiences related to connectivity, platform availability, payment practices, travel, and access conditions. Their occurrence helps situate the experiences discussed in the focus groups but should not be interpreted as evidence of broader geographic patterns or the global expansion of sharing economy platforms.

Regarding organizations (ORG), the most frequently mentioned names included Booking, Airbnb, Cabify, Uber, and BlaBlaCar. Participants referred to these platforms as concrete examples when discussing registration, navigation, payments, trust, accessibility, and customer support. Their recurrence helps link the qualitative findings to the specific platforms discussed in the focus groups, without implying market leadership, broader brand recognition, or popularity beyond the study corpus.

The recurrence of these company names identifies the platforms that were most prominent in this particular corpus. More broadly, the LOC and ORG classifications provide a descriptive overview of the organizations and geographic contexts referenced by participants, without supporting broader conclusions beyond the focus group discussions.

Overall, the NLP analysis provided a complementary descriptive layer to the qualitative findings by making recurring words, lemmas, platform names, and geographic references more visible. These patterns highlighted recurring language associated with usability, technology, skills, trust, and other conditions of use across the transcripts. Nevertheless, the interpretation of the findings remained grounded in the Gioia analysis, participants’ quotations, and the contexts in which their statements were made.

## 5. Discussion

This study provides an exploratory account of how a small group of residents of Madrid, Spain, perceived and experienced digital inequalities when using SE platforms. The five dimensions identified through the Gioia methodology—usability, technology, digital skills, trust, and other usage limitations—provide a useful framework for organizing the barriers described by participants. Given the size and composition of the sample, these dimensions should not be interpreted as a comprehensive or representative account of all platform users and nonusers in Madrid or Spain. Rather than operating as isolated obstacles, the findings suggest that these dimensions may interact, with limitations in one area potentially intensifying difficulties in others. This section discusses each dimension in relation to the existing literature and considers the broader implications of their interaction.

### 5.1. Usability

Participants described several basic platform functions as intuitive and user-friendly, including requesting a ride, booking accommodation, and filtering results. Within this sample, these accounts suggest that many platforms provide relatively accessible entry points for routine tasks. However, as platforms expand into multiservice ecosystems that combine transportation, marketplaces, and financial services, their use may become more demanding, requiring users to adapt to new features and increasingly complex interfaces. This evolving environment is consistent with van Dijk’s [25] characterization of the second-level digital divide as dynamic: narrowing an initial skills gap does not necessarily ensure continued ease of use because technologies, and the demands they place on users, continue to evolve.

Social influence also emerged as an important factor shaping decisions to adopt or avoid a platform. Recommendations from peers and the positive or negative experiences of others appeared to influence participants’ willingness to try a particular service. These accounts suggest that usability is not assessed solely through direct interaction with an interface but may also be shaped by the experiences and opinions circulating within users’ social networks. However, this small qualitative sample does not allow the relative influence of peer recommendations, advertising, and interface design to be determined. This finding should therefore be considered exploratory.

### 5.2. Technology

Participants generally perceived that barriers associated with the first-level digital divide, particularly limited internet access, have become less restrictive in urban areas of Spain because of the wider availability of public Wi-Fi and more affordable mobile data plans. However, these perceptions should be interpreted cautiously because all participants were residents of Madrid, and the study did not directly include rural or peri-urban users. The discussions nevertheless suggested that improvements in urban connectivity may coexist with persistent territorial inequalities, particularly in areas where network coverage and service availability remain limited. In such contexts, sharing economy platforms may be difficult or impossible to access rather than merely less attractive to potential users. This interpretation is consistent with Lutz et al. [39], who identified differences between urban and rural areas in the perceived benefits of the sharing economy and noted that limited service availability may itself constitute a barrier in some rural contexts.

Participants also referred to individuals who may be unable to afford smartphones or mobile data plans. These accounts suggest that economic constraints can create barriers at the level of basic access, before users encounter difficulties related to digital skills, platform usability, or trust. However, individuals experiencing the most severe forms of material and digital exclusion were not represented in the sample. This interpretation is consistent with Dillahunt et al. [31], who showed how limited access to digital devices and internet connectivity can result in exclusion from platform-based services.

### 5.3. Skills

Participants described age-related differences in how individuals approached and navigated digital platforms. Younger users were generally perceived as more familiar with digital technologies and more likely to find platform use intuitive, whereas older users were described as potentially requiring more time, support, or assistance. However, adults aged 65 or older were not represented in the sample. These observations should therefore be interpreted primarily as participants’ perceptions and accounts involving relatives or acquaintances rather than as direct evidence from older platform users. This pattern is consistent with Lutz et al. [9], who identified digital skills as an important predictor of participation in the sharing economy, and with Hidalgo et al. [24], who reported that age and educational attainment were key predictors of digital skills gaps in Spain.

The discussions also highlighted self-directed learning through experimentation, trial and error, and gradual adaptation as an important way in which participants developed digital competence. Rather than showing that informal learning is more effective than formal digital literacy interventions, the findings suggest that experiential learning played a prominent role in this particular sample, which included a relatively high proportion of participants with higher educational attainment and professional experience. For individuals with lower educational attainment, limited digital access, or less confidence in using technology, however, self-directed learning may be more difficult and may not provide adequate support. At the same time, the ongoing evolution of SE platforms may make previously acquired skills less sufficient over time, creating a continuous need for adaptation that may place greater demands on users with less time, confidence, or access to assistance.

Younger participants also suggested that frequent use or apparent ease of use did not necessarily imply a deeper understanding of how platforms operated. Some accounts indicated that users could complete routine tasks without fully understanding how personal data, payments, permissions, or security settings were managed. This distinction suggests that operational familiarity and critical digital competence should not be treated as equivalent. Even users who navigate platforms with ease may remain exposed to risks that they do not fully recognize or assess.

### 5.4. Trust

The security of personal data, the risk of payment fraud, and the perceived vulnerability of some groups, particularly older adults, were recurring concerns in the discussions. Participants described how their fear of fraud had increased over time as they became more aware of negative experiences involving friends, relatives, or others within their social networks. In several cases, this concern appeared to be shaped not only by direct experience but also by accounts shared by people around them. These findings suggest that trust in SE platforms may be influenced by socially shared perceptions of risk as well as by personal experience.

The analysis also identified several precautionary strategies that participants used to manage these concerns, including using cards with low credit limits for online payments, avoiding public Wi-Fi networks when conducting financial transactions, and limiting their use to familiar or well-established platforms. Within this sample, these practices suggest that mistrust did not necessarily lead participants to abandon platforms altogether. Instead, some adapted their behavior in ways that allowed them to continue using these services while attempting to reduce the perceived risks.

Finally, participants expressed particular concern about older adults, whom they perceived as potentially more vulnerable to scams and fraudulent practices. They associated this perceived vulnerability with lower levels of digital literacy and greater difficulty distinguishing legitimate requests from suspicious ones. However, these observations were based primarily on participants’ accounts involving parents, relatives, or acquaintances, as adults aged 65 or older were not represented in the sample. The findings should therefore be interpreted as participants’ perceptions of age-related risk rather than as direct evidence from older platform users.

### 5.5. Other usage limitations

Financial exclusion associated with reliance on credit cards emerged as an additional concern in the discussions. Participants noted that some platforms require or strongly favor card-based payments, which may create barriers for individuals who do not have a credit card or have limited access to formal financial services.

Participants also raised concerns about the accessibility of SE platforms for people with visual or hearing impairments. The spontaneous emergence of this issue suggests that accessibility was important to some participants, although it does not demonstrate that such barriers are widespread across platforms or user groups. Moreover, people with visual or hearing impairments were not specifically recruited for the study. These observations should therefore be interpreted as participants’ perceptions of potential design limitations rather than as firsthand evidence from affected users.

Finally, the quality of customer support, particularly the reliance on automated chatbots, was described as another source of difficulty. Participants suggested that users who encounter complex problems may also be those who most need clear and responsive assistance. Within this sample, automated support systems were often perceived as frustrating or ineffective when they failed to resolve specific issues or provide access to a human representative. In such situations, inadequate support may intensify uncertainty and mistrust, especially when users are already dealing with payment, security, or access-related concerns.

### 5.6. Interconnection of dimensions

An important finding of this study is that the five identified dimensions do not appear to operate independently. Instead, the focus group discussions suggest that difficulties in one area may intensify barriers in others. For example, participants indicated that limited digital skills may make it more difficult to assess platform security, recognize potential risks, or manage privacy settings, thereby increasing trust-related concerns.

Participants also perceived that limited connectivity in rural areas could reduce opportunities for frequent platform use and, consequently, for developing digital familiarity through continued practice. However, rural residents were not represented in the sample, so this relationship should be interpreted as an exploratory proposition rather than as direct evidence from rural users. Similarly, participants noted that the absence of a credit card may restrict access to SE services regardless of an individual’s digital skills or willingness to use them.

The discussions further suggest that barriers may become more difficult to overcome when multiple forms of disadvantage occur simultaneously. This may be particularly relevant for groups such as older adults living in rural areas, individuals with low incomes and lower educational attainment, and people with disabilities. However, the study did not directly examine the experiences of all these groups or the combined effects of these disadvantages.

These findings are consistent with the structural model of digital inequality proposed by Eichhorn et al. [10], particularly in suggesting that interactions among different dimensions may contribute to cumulative disadvantages in platform use. A related argument appears in Lutz et al. [9], who indicate that the demographic and psychological factors associated with participation may operate in interconnected ways.

## 6. Conclusions, limitations and avenues of further research

### 6.1. Conclusions

This study examined how individuals perceive and experience digital inequalities when participating in the sharing economy. Based on data from two focus groups conducted with 14 adults in Madrid in July 2024, the Gioia analysis identified five interrelated aggregate dimensions: usability, technology, digital skills, trust, and other usage limitations. Together, these dimensions suggest that participation depends not only on internet access and digital competence but also on platform design, accessibility, payment options, customer support, and trust in data and transaction security.

The findings suggest that digital exclusion in SE may be cumulative. Individuals may have internet access and some experience with digital technologies yet still face difficulties related to complex interfaces, limited payment options, inadequate customer support, limited digital skills, or concerns about privacy and fraud. The study therefore offers a user-centered framework that links individual digital resources to platform-level conditions and broader socioeconomic constraints.

The Gioia methodology provided the primary interpretive framework for understanding participants’ experiences. NLP was used as a complementary exploratory tool to identify recurring words, platform names, and geographic references in the transcripts. It was not intended to statistically validate the qualitative findings but rather to enhance transparency in the descriptive examination of the corpus.

Because the sample was small, geographically limited to Madrid, and included a relatively high proportion of participants with higher educational attainment or professional experience, the findings should be interpreted as exploratory rather than representative. They may not fully capture the experiences of older adults, rural residents, individuals with lower educational attainment, or those with very limited access to digital technologies. Nevertheless, the findings suggest that more inclusive participation may require attention not only to digital skills and infrastructure but also to simpler interfaces, greater accessibility, flexible payment options, trustworthy data practices, and access to human support.

### 6.2. Implications

The findings have practical implications for platform designers, policymakers, and educators. Although the identified barriers are interconnected, each stakeholder group can address different aspects of digital inequality in participation in the sharing economy.

For platform designers, the findings highlight the need to simplify interfaces and reduce the number of steps required to complete essential tasks. Navigation, registration, identity verification, booking, payment, and cancellation processes should use clear language, visible instructions, and consistent icons. Platforms should also incorporate accessibility features for users with visual, hearing, motor, or cognitive impairments. Greater flexibility in payment methods could reduce barriers for individuals who do not have access to credit cards or who are reluctant to provide financial information online. In addition, access to human customer support should be maintained alongside automated assistance, particularly when users encounter account, payment, security, or service-related problems.

For policymakers, the findings suggest that inclusion in SE requires more than access to a digital device or an internet connection. Public policies should combine investment in affordable and reliable digital infrastructure with initiatives to strengthen digital skills, particularly among older adults, individuals with lower educational attainment, people living in areas with limited connectivity, and other groups at risk of digital exclusion. Policymakers could also encourage platforms to adopt inclusive design principles, accessibility features, transparent data practices, alternative payment methods, and multiple channels for customer support. Where appropriate, regulatory standards could be introduced to promote compliance with these practices. Such measures may help prevent platform-based services from reinforcing existing digital and socioeconomic inequalities.

For educators, training should focus on the practical skills needed to use SE platforms safely and independently. Programs could include guided exercises on creating accounts, navigating platforms, comparing services, making and canceling bookings, completing digital payments, managing passwords, protecting personal data, preventing fraud, and accessing customer support channels. Training should be tailored to participants’ prior experience and level of confidence rather than assuming a uniform level of digital competence. Libraries, adult education centers, universities, local associations, and community organizations may be particularly well positioned to provide personalized support and practice-based learning in trusted settings.

### 6.3. Limitations and avenues of further research

This study has several limitations that should be acknowledged. First, its geographic focus on Madrid, Spain, together with the relatively small sample size, limits the generalizability of the findings. Although the qualitative approach provides in-depth insight into participants’ experiences and perceptions, the findings may not capture the cultural, social, and economic variations present in other contexts.

In addition, the sample was limited to adults aged 25–64 and therefore did not include the direct perspectives of older adults. Insights related to this group were based on participants’ accounts involving relatives, which limited the extent to which older adults’ experiences could be represented in the analysis. The sample also included a relatively high proportion of participants who had completed or were pursuing higher education. Consequently, the experiences and challenges of individuals with lower educational attainment were not directly captured.

Future research could broaden the scope of this study by comparing different countries or regions to examine how the identified dimensions vary across cultural, social, and economic contexts. Longitudinal studies could also explore how users’ perceptions and digital skills evolve over time. In addition, quantitative research could complement the qualitative findings and assess the extent to which the observed patterns apply to larger and more diverse populations. Social media sentiment analysis could provide a further exploratory perspective on how different population groups discuss and perceive the use of SE platforms.

## Supporting information

S1 File Focus group discussion guide. (DOCX)
