# Supplementary material for: Digital inequalities in the sharing economy: A qualitative text analysis from focus groups
Source: PLoS One. 2026 Sep 11;21(9):e0357784. doi: 10.1371/journal.pone.0357784 (PMC13567767; doi:10.1371/journal.pone.0357784)
Supplement: Supplementary file 1 [file pone.0357784.s001.docx]

**S1. Focus group discussion guide**

**Facilitator:** David Coy-Mesa, PhD candidate, Department of Organization Engineering, Business Administration, and Statistics, Universidad Politécnica de Madrid. Gender: Male. Experience: Completed a Market Research course and trained in qualitative methods and focus group moderation.

**Observer:** PhD. Gustavo Morales-Alonso, co-author, Department of Organization Engineering, Business Administration, and Statistics, Universidad Politécnica de Madrid. Gender: Male. Experience: Note-taking and group dynamics observation.

**Pilot testing:** The guide was pilot-tested in a preliminary session to refine questions and timing.

**Instructions:** Sessions lasted 50-60 minutes. Questions were open-ended to explore participants' experiences freely. Audio-recorded with informed consent. Field notes were taken by the observer.

**Structure (4 blocks):** The focus group discussions were organised into four thematic blocks aligned with the research question and propositions. Each session lasted approximately 50–60 minutes and was guided using open-ended questions designed to explore participants’ experiences and perceptions regarding digital skills, access barriers, and engagement with SE platforms.

**Block 1. Impact of Digital Skills (15 minutes)**

- How have your digital skills influenced your ability to access and benefit from sharing economy platforms?

**Block 2. Access to the Sharing Economy and Digital Divide (15 minutes)**

- In your opinion, how do digital access barriers affect individuals with fewer economic resources in their use of sharing economy platforms? Please describe any specific experiences you are aware of.

**Block 3. Social and Economic Implications (15 minutes)**

- How do you think digital skills influence your participation in the sharing economy? For example, in terms of internet navigation, use of social media, or use of digital applications.
- What difficulties have you encountered when using these platforms, particularly in relation to limited digital skills or lack of internet access?

**Block 4. Proposals and Improvements (15 minutes)**

- Based on your experience, what improvements would make these platforms more inclusive and accessible?

**Closing (5–10 minutes)**

- Final round of comments.
- Acknowledgements and closing remarks.
